# Loss of XBP1 accelerates age-related decline in retinal function and neurodegeneration

**DOI:** 10.1186/s13024-018-0250-z

**Published:** 2018-04-04

**Authors:** Todd McLaughlin, Marek Falkowski, Jae Whan Park, Stephen Keegan, Michael Elliott, Joshua J. Wang, Sarah X. Zhang

**Affiliations:** 1 Departments of Ophthalmology and Biochemistry, Ross Eye Institute, University at Buffalo, State University of New York, 3435 Main Street, Buffalo, NY 14214 USA; 2 SUNY Eye Institute, State University of New York, Buffalo, NY USA; 3 Departments of Ophthalmology and Physiology, Dean McGee Eye Institute, University of Oklahoma Health Sciences Center, Oklahoma City, OK USA

**Keywords:** X-box binding protein 1, Aging, Retina, Neurodegeneration, Unfolded protein response

## Abstract

**Background:**

Aging is the strongest risk factor for neurodegenerative diseases and extended age results in neuronal degeneration and functional decline in the visual system. Among many contributing factors to age-related deterioration of neurons is an insufficient activation of the Unfolded Protein Response (UPR) in the endoplasmic reticulum (ER) in response to cellular stress. X-box binding protein 1 (XBP1) is a major component of the UPR and is essential for maintaining protein homeostasis and reducing cellular stresses. Herein, we investigate the role of XBP1 in maintaining morphological and functional integrity in retinal neurons during adulthood and the early stages of aging.

**Methods:**

The basal and induced levels of XBP1 activation in the retina were measured in young adult and aged mice. Conditional knockout (cKO) of XBP1 in retinal neurons was achieved by crossing XBP1 floxed mice with a retina specific Cre-recombinase line (Chx10-Cre). Retinal morphology, neuronal populations including photoreceptors, bipolar cells, and retinal ganglion cells (RGCs), synaptic structure, and microglial activation were examined with immunohistochemistry and staining of retinal sections. Retinal function was evaluated with light-adapted (photopic) and dark adapted (scotopic) electroretinograms. Retinal mitochondrial function and metabolism was assessed by Seahorse XF^e^24 Extracellular Flux Analyzer.

**Results:**

The retinas of aged wild type (WT) mice display a significantly reduced basal level of Xbp1s and compromised activation of ER stress response. In XBP1 cKO mice, significant structural degeneration of the retina, evidenced by thinning of retinal layers and a loss of RGCs, and functional defects indicated by diminished photopic and scotopic ERG b-waves are observed at the age of 12–14 months. Furthermore, discontinuous and disorganized synaptic laminae, colocalized with activated microglia, in the inner plexiform layer is found in the XBP1 cKO retinas. In addition, cKO mice demonstrate a significant increase in ectopic synapses between bipolar cells and photoreceptors, which is strikingly similar to WT mice at 20–24 months of age. These changes are associated with defective retinal glycolysis while mitochondrial respiratory function appears normal in the cKO retina.

**Conclusions:**

XBP1 cKO mice at 12–14 months of age show significant structural, functional, and metabolic deficits that closely resemble WT mice twice that age. Our findings suggest that the absence of XBP1, a critical component of the UPR, accelerates age-related retinal neurodegeneration.

**Electronic supplementary material:**

The online version of this article (10.1186/s13024-018-0250-z) contains supplementary material, which is available to authorized users.

## Background

In mammals, age-related deterioration of the sensory nervous system is common, while repairing or replacing the injured or dead neuronal cells is difficult due, in part, to the lack of cell proliferation and differentiation after development. Thus, the functionality of sensory systems is dependent on the long-term viability of terminally differentiated cells. Mice in captivity can live for three years, however, by about two years of age, the retina has become significantly thinner and less responsive in the electroretinogram (ERG) than in mature, but much younger, adult mice (e.g. 4 months) [1]. Additionally, bipolar cells in aged retinas extend dendrites into the outer nuclear layer (ONL) to form ectopic synapses with retracted rod axons [2, 3]. These changes are recapitulated in mice with deletions of either LKB1 or AMPK, key metabolic pathway controlling proteins, in subsets of retinal cells at just two months of age [4]. These findings indicate that disturbance in retinal metabolism is likely behind some age-related deterioration in retinal neurons. However, the exact mechanisms remain elusive.

Recently, several reports demonstrate a role for endoplasmic reticulum (ER) stress in aging and neurodegenerative diseases, though the mechanisms remain elusive due to seemingly contradictory results across species and tissues [5, 6]. ER stress occurs in a cell when there is an abundance of unfolded or misfolded proteins in the ER. To ameliorate ER stress, three pathways of the Unfolded Protein Response (UPR), namely IRE1, ATF6, and PERK, are activated. These pathways work in parallel to reestablish the ER’s homeostasis through increasing the folding capacity of the ER, slowing protein translation, and increasing protein degradation [7]. However, in scenarios where ER stress is too severe or endures for too long that homeostasis cannot be restored, the UPR can shift to a pro-apoptotic mode promoting cell death, often through increased CHOP expression [6]. X-Box binding protein 1 (XBP1) is the main effector of the IRE1 pathway, playing a crucial role in cell adaptation to ER stress. XBP1 is activated via a unique RNA splicing event initiated by IRE1. Spliced XBP1 (XBP1s) encodes an active transcription factor that regulates a diverse set of genes including ER chaperones and proteins involved in ER-associated degradation (ERAD) that facilitate the restoration of the homeostasis of the ER [7]. Long-term or extreme stress, however, can result in differential oligomerization of IRE1, resulting in a distinct array of RNA splicing targets that tilt the cell towards apoptosis and away from XBP1 splicing [8]. Systemic ablation of XBP1 causes embryonic lethality [9]. Conditional depletion of XBP1 in the nervous system results in impairment in learning and memory formation, suggesting that XBP1 may play a role in controlling neuronal function [10]. In addition, the UPR and XBP1 in particular have been shown to regulate longevity. For example, activation of the UPR, including XBP1, or neuronal expression of XBP1s extends lifespan in *C. elegans* [11, 12]. Long-term intranasal injection of exogenous Hsp70 (an ER chaperone) also delays age-related deterioration and improves learning and memory in aging mice [13]. These studies suggest that successful adaptation to cellular stress through the UPR, and potentially XBP1, is critical for maintaining neuronal viability as well as the integrity of sensory systems as the organism ages. Though the literature is inconsistent across species and tissues, there is strong evidence that the ability to activate the UPR declines with advanced age suggesting a role for ameliorating cellular stresses in longevity and potentially implicating long-term ER stress as a causative aging factor.

In the present study, we identified that aged retinas have a compromised ER stress response with reduced levels of XBP1s under both basal and stressed conditions. To elucidate the role of XBP1 in retinal neuronal maintenance especially during aging, we generated a retina-specific conditional knockout (cKO) mouse line of XBP1 and characterized the retinal structure and function of the mice from an early age to over one year old. Our data suggest that age-related neurodegeneration occurs at an accelerated pace in the retina lacking XBP1 thus indicating a critical role of XBP1 in regulation of age-related retinal degeneration.

## Methods

### Animals and genotyping

Conditional knockout of XBP1 in the retina was achieved by crossing mice with LoxP sites flanking exon 2 of XBP1 [14] with a retina-specific Chx10-Cre line [15]. The two independent lines have been interbred and maintained as XBP1 fl/fl and XBP1 fl/fl; Chx10-cre for multiple generations over several years. Genotyping was performed by PCR with allele-specific primers for XBP1 WT, floxed, and exon 2-deleted alleles. PCR using the primers 3’lox-S - 5’-ACT TGC ACC AAC ACT TGC CAT TTC-3′ and 3’lox-A - 5′- CAT TAC AGG CAG TGA ACC ACC TTG-3′ result in a 140 bp band for WT and a 180 bp band for XBP1-fl. The XBP1-fl allele after Cre-mediated recombination is detected with Int1-S: 5′ - CTT TGT GGT CGT AGG GTA GGA ACC - 3′ and 3’lox-A, resulting in a 352 bp band after recombination, and no detectable band in WT or non-recombined XBP1-fl. The presence of the Chx10-Cre allele was determined by PCR with Cre-F 5′- GCA TTA CCG GTC GAT GCA ACG AGT GAT G-3′ and Cre-R 5′- GAG TGA ACG AAC CTG GTC GAA ATC AGT G - 3′, resulting in a 408 bp band.

### Ex-vivo retinal explant cultures and reverse transcription quantitative PCR (qPCR)

Retinas were dissected and one half cultured for 6 h in Neurobasal-A medium supplemented with 25uM glutamic acid, 2 mM glutamine, and B-27 (all ThermoFisher) and treated with DMSO as control and the other half retina cultured in identical media with 5 μm thapsigargin (ThermoFisher). Retinas were washed in PBS and RNA isolated with Trizol per manufacturer instructions. Complementary DNA (cDNA) was made using Bio-Rad cDNA kit by manufacturer instruction and 500 ng of total RNA. Reverse transcription-qPCR was performed using Bio-Rad iQ Sybr Green Supermix by manufacturer instruction with 10 ng of cDNA, and 300 nM of each of the following primers and normalized to 18 s.: XBP1sF 5’-CCA TCA CAT TGC CTA GAG GAT A-3′ and XBP1R 5’-AGC TGA GTG TCA AAC GAC AAT A-3′; ATF4F 5’-CCC CCT TCG ACC AGT CGG GT-3′ and ATF4R 5’-CCG CCT TGT CGC TGG AGA ACC-3′; ATF6F 5’-CAG ACT CGT GTT CTT CAA C-3′ and ATF6R 5’-GGC TTC TCT TCC TTC AGT-3′; p58ipkF 5’-TCC TGG TGG ACC TGC AG TACG-3′ and p58ipkR 5’-CTG CGA GTA ATT TCT TCC CC-3’ CHOPF 5’-GTC CCT AGC TTG GCT GAC AGA-3′ and CHOPR 5′-TGG AGA GCG AGG GCT TTG-3′; 18 sF 5′-GTA ACC CGT TGA ACC CCA TT-3′ and 18 sR 5’-CCA TCC AAT CGG TAG TAG CG-3′.

### Electroretinography (ERG)

Visual function was assessed by dark- and light-adapted electroretinogram (ERG) using a Diagnosys Espion ColorDome system and software (Diagnosys LLC, Lowell, MA). For photopic transient ERG the manufacturer installed Light plus OPs protocol was used. Mice were anesthetized with intraperitoneal injection of 120 mg/kg ketamine and 5 mg/kg xylazine, and had pupils dilated with 1% atropine (Falcon Pharmaceuticals) followed by 2.5% Phenylephrine Hydrochloride (Bausch & Lomb). Mice were placed in the ColorDome apparatus, an electrode was inserted into the tail as ground, and a reference electrode placed subcutaneously between the eyes. Gonak (Akorn) ophthalmic gel was applied liberally to each cornea, and wire electrodes placed in contact. Animals were light adapted for a minimum of 15 min prior to stimulation and subjected to five flashes of 4 ms duration at 1 Hz at 10 cd.s/m^2^ with a background of 5 cd.s/m^2^. The a-wave amplitude was recorded as the lowest point of the initial response compared to baseline, and the b-wave amplitude was the high point, as calculated from the a-wave amplitude. The average value for both eyes for all flashes is reported.

For the dark-adapted step ERGs, a custom procedure was designed within the Diagnosys software. Briefly, mice were dark adapted overnight (14–16 h) in their home cage. Under dim red light, mice were anesthetized, dilated, and set in the ColorDome as described above. A protocol consisting of ten series of three flashes of light of 4 ms duration was applied. Light intensity increases with each subsequent series. There was a delay between each flash of 15–60 s, with the delay increasing with intensity. Light intensities were (luminance in log(cd.s/m^2^)): − 3.6, − 3.0, − 2.4, − 1.8, − 1.2, − 0.6, 0.0, 0.6, 1.4, 2.1. Amplitudes for all traces from both eyes were averaged for each light intensity and animal. The reported N value represents independent animals. The peak for the a-wave was the lowest point of the initial response to the flash, and the b-wave peak was determined to be the peak after the oscillatory potentials and within 140 ms of the a-wave, and its magnitude measured from the a-wave peak. The average value for both eyes for all flashes is reported. For ERG on 10 week old animals the average age for WT was 10.8+/− 1 weeks and 10.4+/− 1.2 weeks for XBP1 cKO mice. For the 6–8 month cases WT average age was 32.6 +/− 3 weeks and 29.7+/− 4 weeks for XBP1 cKO mice. For 12–14 month ERGs WT average age was 62.3+/− 2 weeks old and XBP1 cKO average age was 59.1+/− 5 weeks old.

### Immunohistochemistry and retinal staining

Retinal morphology was examined with immunohistochemical markers in WT and XBP1 fl/fl; Chx10-Cre in retinal sections in adolescent and 12–15 month old mice (average ages for morphological analyses was 59.2+/− 5 weeks for WT and 56.1+/− 5 weeks for XBP1 cKO mice). Eyes were immersion fixed in 4% *w*/*v* paraformaldehyde in phosphate buffered saline (PBS), pH 7.4 for one hour, washed, and equilibrated in 30% sucrose w/v in PBS at 4C. Eyes were embedded in OCT and frozen in molds partially submerged in absolute ethanol chilled on crushed dry ice. Blocks were cryosectioned immediately or stored at -80C. Cryosections were cut at -20C at 20 μm, mounted on Superfrost Plus slides (Statlab), and dried overnight.

Sections were blocked with PBS plus 1% triton X-100 plus 1% BSA fraction V (Calbiochem) for 1 h and incubated with primary antibody overnight at 4C in a light-protected, humidified chamber. Primary antibodies were removed, sections washed with PBS plus 1% Triton X-100, and incubated with the appropriate secondary antibody in block solution for 1 h in a light-protected, humidified chamber. Secondary antibody was washed and sections mounted with Vectashield mounting medium with DAPI (Vector, H-1200) for examination. All antibodies used are listed in Table 1.

**Table 1:** Antibodies used in immunofluorescence (IF) and western blot analyses (WB)

Antibody	Dilutions	Catalog No.	Company
anti-Ribeye, B-domain	1:800 (IF)	192,003	Synaptic Systems
anti-Pkc-α	1:400 (IF)	sc-8393	Santa Cruz Biotechnology
anti-Calretinin, clone 6B8.2	1:800 (IF)	Mab1568	Millipore
anti-Iba1	1:800 (IF)	019–19,741	Wako
anti-XBP1	1:1000 (WB)	sc-7160	Santa Cruz Biotechnology
anti-Glutamine synthetase	1:800 (IF)	Mab302	Millipore
anti-Brn3a	1:800 (IF)	sc-31,984	Santa Cruz Biotechnology
anti-Pax6	1:40 (IF)	Pax6-s	DSHB
anti-β actin	1:10,000 (WB)	ab8226	Abcam
Peroxidase	1:10,000 (WB)	PI-2000	Vector
Peroxidase	1:10,000 (WB)	PI-1000	Vector
Texas Red	1:800 (IF)	T6391	Molecular Probes
Alexa Fluor-488	1:800 (IF)	A11001	Molecular Probes
Alexa Fluor-594	1:800 (IF)	A11005	Molecular Probes
Alexa Fluor-594	1:800 (IF)	A11080	Molecular Probes
anti-Calbindin D-28 K, CB-955-955	1:100 (IF)	Sab4200543	Sigma

Cryosections of retina were incubated in 1-Step NBT/BCIP alkaline phosphatase labeling reagent (Thermo Scientific, #34070) until an appropriate chromogenic signal was observed.

Photographs were taken with an Olympus DP80 digital camera mounted on an upright Olympus BX53 microscope using 10-40× objectives, processed, montaged, and analyzed with Photoshop (Adobe) and NIH ImageJ software.

### Western blot analysis

Retinas were dissected and flash frozen in liquid nitrogen. Tissue was thawed on ice in chilled fresh radio immune precipitation assay (RIPA) buffer with protease inhibitor mixture, PMSF, and sodium orthovanadate and sonicated. Protein samples (approximately 25 μg), MagicMark XP Western Protein Standard (Life Technologies, LC5602), and BenchMark Prestained Protein Ladder (Life Technologies, 10,748–010) were electrophoresed in 10% acrylamide resolving gels and transferred to nitrocellulose blotting membranes using standard techniques. Membranes were incubated with anti-XBP1 (Santa Cruz Biotechnology) at 1:1000 in Tris-buffered saline plus tween overnight at 4C. followed by incubation with peroxidase conjugated secondary antibody (Vector), at 1:10000 for 1 h room temperature before development with SuperSignal West Dura Extended Duration Substrate (Thermo Scientific, #34076) per manufacturer instructions. Blots were imaged and analyzed with a Bio-Rad ChemiDoc MP imaging system using ImageLab software. Bands were re-blotted with anti-beta-actin (Abcam 1:10000, overnight at 4C) and then peroxidase conjugated secondary antibody as above, as loading control.

### Metabolic measurements

Retina from 12 to 14 month WT or XBP1 fl/fl; Chx10-Cre mice was dissected into 15–20 explants of nearly equal size and plated in a prepared 24 well assay dish, GCL side down. A single 24 well assay dish was used per experiment and contained ten explants each from a WT retina and an age-matched XBP1 cKO retina. Five explants of each genotype were subjected to glycolytic testing and five explants of each genotype were used for mitochondrial testing in a Seahorse XF^e^24 Extracellular Flux Analyzer using manufacturer supplied kits. The glycolysis stress test used final well concentrations of 10 mM glucose, 1 μM oligomycin, and 50 mM 2-D-glucose. Compounds were injected just after measurement steps 3, 6, and 9, respectively. For the mitochondrial stress test, FCCP was injected just after measurement 3 to a well concentration of 1 μm and rotenone/antimycin A was injected after measurement 9 to a well concentration 0.5 μM. Readings for ECAR and OCR were taken at each measurement interval. Immediately after the final measurements each explant was removed from the dish, centrifuged briefly, assay media was removed, and RIPA lysis buffer added. Tissue was then sonicated and subjected to BCA protein assay according to the manufacturer protocol (Thermo Scientific). Measurements from each well were normalized to total protein content of the corresponding explant. Average age of WT mice used for metabolic measurements was 58.2+/− 5 weeks and the average age of XBP1 cKO mice used was 59.1+/− 5 weeks old.

### Data analysis

RGC cell counts were performed automatically in NIH Image J using the Analyze Particles macro modified to accurately count cells in single-channel images of Brn3a immunohistochemistry using secondary antibodies conjugated to Texas Red and of single channel images of DAPI labeling. Counting was restricted to the GCL layer of cryosections from central retina containing the optic disc, or adjacent, and containing at least the full extent of the GCL from central retina to the periphery. Areas where the plane of section was tangential were excluded. Between 3 and 5 sections per case were counted blind to genotype and averaged. Automated counting paradigms were verified by comparisons to a subset of manually counted images by a second operator. Linear distances were measured through the GCL layer.

Calretinin-labeled synaptic lamina data was assessed by two independent investigators blind to genotype and the presence or absence of an Iba1-positive cell. Areas analyzed were chosen without knowledge of calretinin staining by random selection of Iba1-positive cells A score of ‘continuous’ or ‘discontinuous’ was applied to each case from a photograph of the IPL with calretinin staining. Assessments were made using agreed-upon criteria, and repeated at a later date with re-blinded cases. Assessments were approximately 90% identical between investigators and between assessments.

Retinal layers were measured in photographs of DAPI-stained 20 μm cryosections by at least two independent investigators blind to genotype. Measurements were taken in NIH ImageJ from retinal sections from the central 15% of retina encompassing the optic disc, cut orthogonal to the pupillary plane, and containing at least the full extent of all retinal layers from central retina to the periphery. Layers were measured at two locations approximately 300 μm from the optic disc (or linear center of sections adjacent to the optic disk) in each direction.

Data were compiled and analyzed in Microsoft Excel. Figures and photos used for data analyses were montaged and assembled in Adobe Photoshop, NIH Image J, and/or Microsoft PowerPoint.

### Statistical analysis

Statistical analyses were performed in Microsoft Excel. Statistical analyses were unpaired Student’s t-test when comparing two groups. Two-way ANOVA with Bonferroni post-hoc test was used for the light adapted and dark adapted ERG analyses, layer thickness measurements, and glycolysis test ECAR measurements. Statistical differences were considered significant at a *P* value less than 0.05. N values represent independent animals except where noted and all experiments were performed independently at least twice. Error bars indicate standard deviation except where noted.

## Results

### Compromised ER stress response in the retina of aged wild type mice

**Fig. 1:**
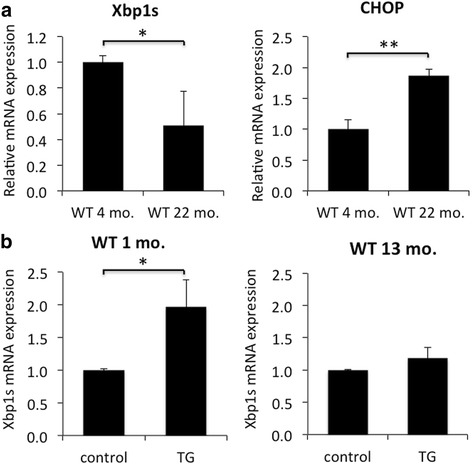
Reverse-transcription quantitative PCR reveals aged mice have a compromised ER stress response. **a** Reverse-transcription quantitative PCR (qPCR) was performed on total RNA extracted from whole retina from 4 month old (*n* = 4) and 20–24 month (*n* = 3) old XBP1 fl/fl (WT) mice. Primers specific for spliced Xbp1 (Xbp1s) reveal a 50% reduction in mRNA level in aged retina. Conversely, CHOP mRNA is significantly elevated in aged retina compared to young adult. **b** Graph of relative mRNA expression of XBP1s using RNA extracted from WT retinal explants subjected to vehicle or 5 μm thapsigargin (TG) for 6 h ex vivo. Retina from one month old mice (*n* = 3) respond to TG with a significant 2-fold increase in XBP1s. In contrast, retina from 13 month old mice (*n* = 3) have a minor 18% increase in XBP1s expression. All qPCR data normalized to 18 s. *, *p* < 0.02; **, *p* < 0.001

We examined the mRNA level of UPR genes including XBP1s, ATF4, ATF6, p58ipk, and CHOP in young adult (4 months old) and aged (20–24 months old) XBP1 fl/fl (wild type, WT) mouse retina using reverse transcription quantitative PCR (qPCR). We find a significant 50% decrease in the level of XBP1s in aged WT retina compared to young WT retina (Fig. 1a). In contrast, there is a significant, nearly 2-fold, increase in CHOP mRNA level in aged WT retina compared to young WT retina (Fig. 1a). We find a trend for lower levels of ATF4 (25% decrease, *p* > 0.4), ATF6 (10% decrease *p* > 0.75), and p58ipk (25% decrease, p > 0.4) but these do not reach statistical significance. These results suggest that the UPR of aged retina is more biased towards the CHOP pathway, generally associated with apoptosis, than the XBP1s pathway, generally associated with maintaining the ER homeostasis.

In addition, we assessed the ability of the young and old WT retina to respond to ER stress using an ex vivo retinal explant culture system (see Methods). We find that thapsigargin (TG) treatment, which elicits ER stress response, results in a 2-fold increase in XBP1s mRNA level compared to control in 1 month-old WT retinal explants. In contrast, identical treatment of 13 month-old WT retina fails to induce a significant increase in XBP1 activation (Fig. 1b). These data provide further evidence suggesting that aged retina has a compromised ER stress response.

### Depletion of XBP1 does not affect retinal neuronal development

**Fig. 2:**
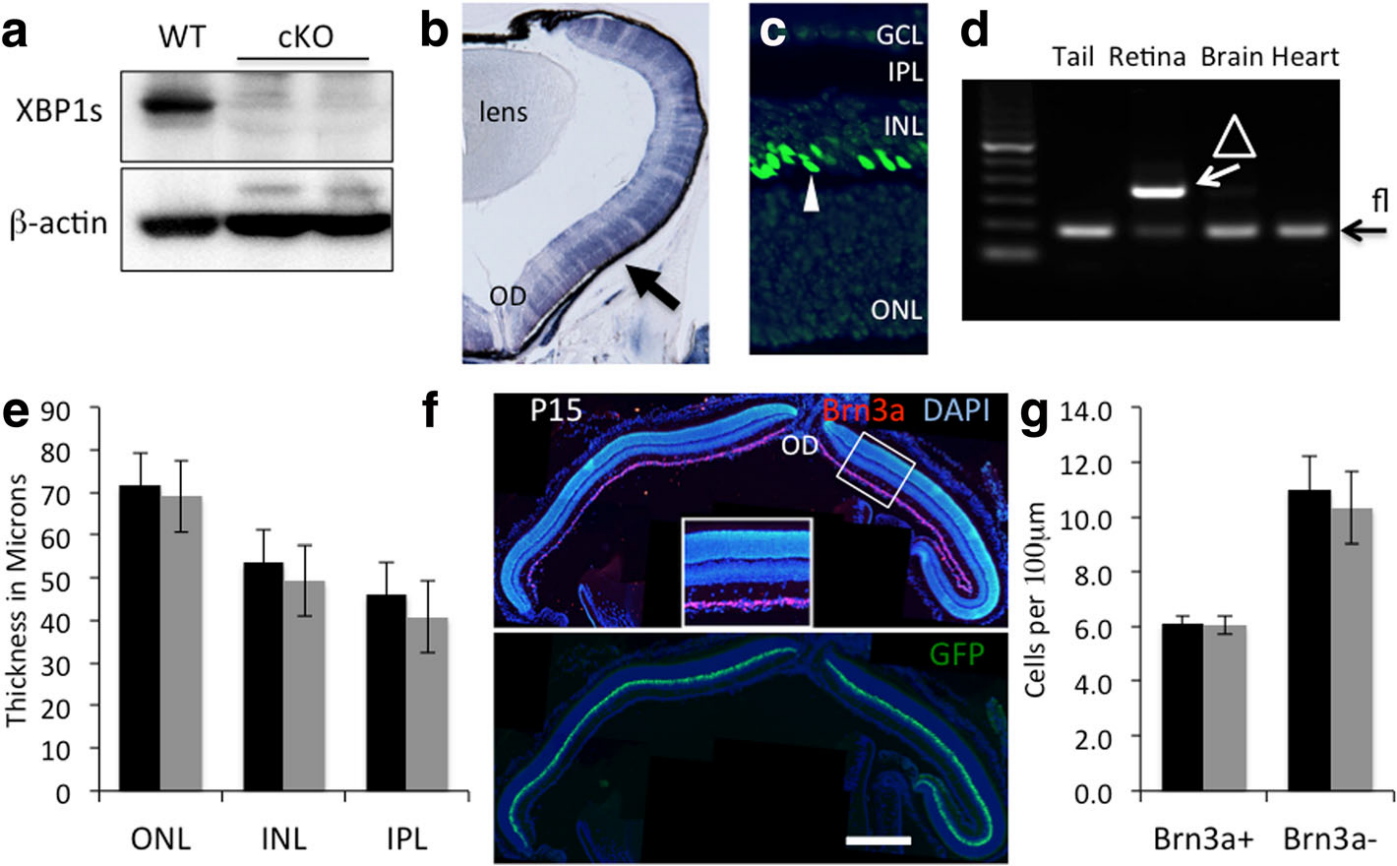
Conditional knockout of XBP1 in the retina through the use of Chx10-Cre does not affect retinal development. **a** Western blot of whole retina at P4 reveals a significant decrease is XBP1s protein in XBP1 f/f; Chx10-cre (cKO) retina, compared to WT. **b** Cryosection of P4 retina after alkaline phosphatase staining (blue, arrow) reveals Cre-recombinase activity in all retinal layers, but not all retinal cells, at this age. **c** Cryosection through P15 retina counterstained with DAPI demonstrating that the Chx10 GFP-Cre recombinase fusion protein is localized to bipolar cells at this age (arrowhead). **d** PCR with primers specific for the non-recombined XBP1 floxed (fl) and recombined (Δ) XBP1 alleles reveals that Cre-mediated recombination is specific to retina. **e** Graph of measurements of retinal layers at P15 showing no statistically significant differences in the outer nuclear layer (ONL), inner nuclear layer (INL), and inner plexiform layer (IPL) between WT (*n* = 5) and XBP1 cKO (*n* = 7) at this age. **f** Cryosection through central retina of an XBP1 cKO mouse labeled with antibodies against the RGC marker Brn3a (red) and counterstained with DAPI (blue) at P15. Inset is higher magnification view of the boxed area. Lower panel is the same section showing the GFP-cre fusion protein is present in bipolar cells throughout retina at this age. **g** The number of Brn3a-positive and Brn3a-negative cells within the ganglion cell layer (GCL) at P15 are not statistically different between WT (*n* = 5) and XBP1 cKO (*n* = 6). OD, optic disk. Scale bar = 200 μm in B, 50 μm in C, 350 μm in F, and 150 μm in F, inset

To delineate the role of XBP1 in retinal neuronal development and maintenance, we generated a conditional knockout mouse line to delete XBP1 in retinal neural progenitor cells by crossing an XBP1 floxed line [14] with the Chx10-Cre line, in which a Cre-recombinase-GFP fusion protein is expressed in a subset of retinal neurons [15]. Western blot analysis confirmed the knockout efficiency, demonstrating that XBP1 protein is greatly reduced in the retina of conditional knockout XBP1 fl/fl; Chx10-Cre (cKO) mice compared to XBP1 fl/fl (wild type, WT) mice (Fig. 2a). At postnatal day 4 (P4), Cre is expressed broadly across all developing retinal lamina in the XBP1 cKO retina, as illustrated by alkaline phosphatase reporter staining (Fig. 2b). Notably, though broadly expressed, Chx10-cre is not expressed in all retinal cells at this age, resulting in a mosaic pattern across the retina. By the second postnatal week, Chx10-Cre expression is limited to bipolar cells in the retina (Fig. 2c). In addition, we confirmed that Cre expression is limited to the retina as indicated by PCR on DNA extracted from various tissues from adult XBP1 cKO mice using primers specific for the XBP1 floxed allele and for the deleted allele (Fig. 2d).

Since XBP1 deletion occurs during retinal development, we first determined if the XBP1 cKO retina develops normally. By P15 the retina resembles its mature form and major retinal cell types are present at or near their final populations. We examined retinal structure and cell type composition at P15 and find only minor, non-statistically significant differences between XBP1 cKO and WT mice (Fig. 2e-g). We find no significant difference in the thickness of the retina for any individual layer, though the XBP1 cKO retina does trend as slightly thinner overall (Fig. 1e). Furthermore, we find no obvious difference in the numbers of cells labeled with the retinal ganglion cell (RGC) marker, Brn3a (Fig. 1f and g), with the bipolar marker PKC-α, the Müller Glia (MG) marker, GS, or the amacrine cell marker, Pax6 (Additional file 1: Figure S1). Furthermore, within the ganglion cell layer (GCL) we find no difference in the number of cells labeled with the nuclear dye DAPI that are Brn3a-negative (Fig. 1g). These cells represent primarily displaced amacrine cells and Brn3a-negative RGCs, which are a significant minority of all RGCs.

### Accelerated decline in retinal function in XBP1 cKO mice at age 12–14 months

**Fig. 3:**
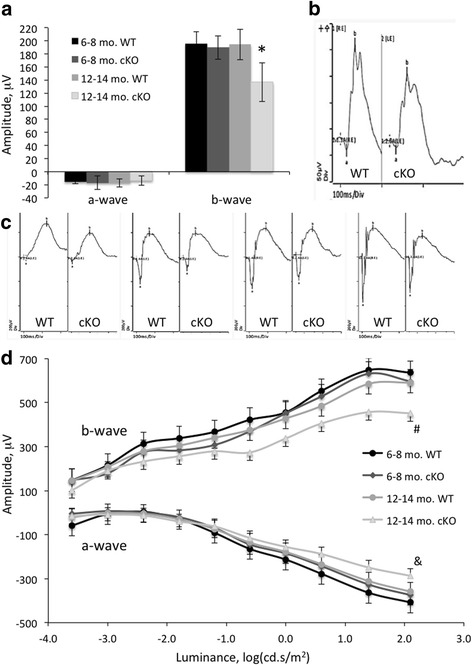
Retinal function declines in XBP1 fl/fl; Chx10-Cre mice after eight months of age. **a** Graph of the a-wave and b-wave responses of a transient, light-adapted ERG in XBP1 fl/fl (WT) mice at ages 6–8 months (*n* = 3) or 12–14 months (*n* = 9) and XBP1 fl/fl; Chx10-Cre (cKO) mice at ages 6–8 months (*n* = 3) or 12–14 months (*n* = 11). There is a significantly reduced b-wave in XBP1 cKO compared to WT mice at age 12–14 months. In addition, there is a significant age-related decline in the b-wave amplitude in XBP1 cKO but not WT mice (*, *p* < 0.001, two-way ANOVA). There is no significant difference in the amplitudes of the a-waves. **b** Traces from light-adapted transient ERG that closely resemble the average responses for WT (left) and XBP1 cKO mice at age 12–14 months. **c** ERG traces closely resembling the average responses for WT and XBP1 cKO for 4 of the 10 stimulus intensities of a 10-step, dark adapted ERG from mice age 12–14 months. Note the similar shape and onset times, but distinct amplitudes in the b-wave and a-wave between genotypes. **d** Graph of the 10-step dark adapted ERG responses in 6–8 month-old (*n* = 3 per group) or 12–14 month-old (*n* = 8 per group) WT and XBP1 cKO mice. At age 6–8 months there is no significant difference in the a-wave or b-wave responses between WT and XBP1 cKO mice. However, at age 12–14 months, there is a significantly diminished a-wave (&, *p* < 0.01, two-way ANOVA) and b-wave (#, *p* < 0.02, two-way ANOVA) in the XBP1 cKO compared to the WT. Furthermore, there is a significant age-related decline in the b-wave amplitude for the five highest light intensities (*p* < 0.03, two-way ANOVA) and in the a-wave amplitude for the three highest light intensities (*p* < 0.01, two-way ANOVA) in XBP1 cKO but not in WT mice

We analyzed retinal function with light-adapted (photopic) ERG at age 2–3 months, 6–8 months, and 12–14 months and dark-adapted (scotopic) ERG in mice at age 6–8 months, and 12–14 months. The photopic and scotopic ERG recordings interrogate the cone and rod photoreceptor pathways, respectively. We find that retinal function is indistinguishable in XBP1 cKO and WT mice at age 2–3 months (Additional file 2: Figure S2) or 6–8 months. As shown in Fig. 3a, the amplitudes of photopic a-wave and b-waves are indistinguishable between XBP1 cKO and WT at 6–8 months of age. In contrast, photopic b-wave amplitude is significantly reduced in XBP1 cKO mice compared to WT controls at 12–14 months of age (Fig. 3a and b). In addition, two-way ANOVA analysis suggests a significant age-related decline in the b-wave amplitude in XBP1 cKO but not WT mice (Fig. 3a). While the amplitude of a-wave is also reduced in XBP1 cKO mice compared to WT, it does not reach statistical significance (Fig. 3a). We find no difference in implicit time between WT and XBP1 cKO responses at any time point examined for light or dark adapted ERG (Additional file 2: Figure S2).

In scotopic ERGs, which were performed at each of ten stimuli over several orders of magnitude of increasing light intensity (Fig. 3c and d), the intensity/response curves of a- and b-waves in XBP1 cKO and WT mice at 6–8 months of age are indistinguishable (Fig. 3d). In stark contrast, we find a significant decline in the amplitudes of both a-wave and b-wave in 12–14 month old XBP1 cKO mice compared to age-matched WT mice (Fig. 3d). ERG recordings show that the shape and implicit times of retinal responses are not different (Fig. 3c and data Additional file 2: Figure S2). However, the amplitudes of the responses are significantly different. In XBP1 cKO, there is a significant age-related decline in b-wave amplitude compared to WT. Similarly, we find a significant age-related decrease in the a-wave response amplitude in the XBP1 cKO compared to WT (Fig. 3d). Thus, between 6 and 8 months and 12–14 months of age, we find a significant age-related decline in the photopic b-wave, scotopic a-wave, and scotopic b-wave in XBP1 cKO, but not in WT mice.

### Advanced deterioration of retinal structure in XBP1 cKO mice at age 12–14 months

**Fig. 4:**
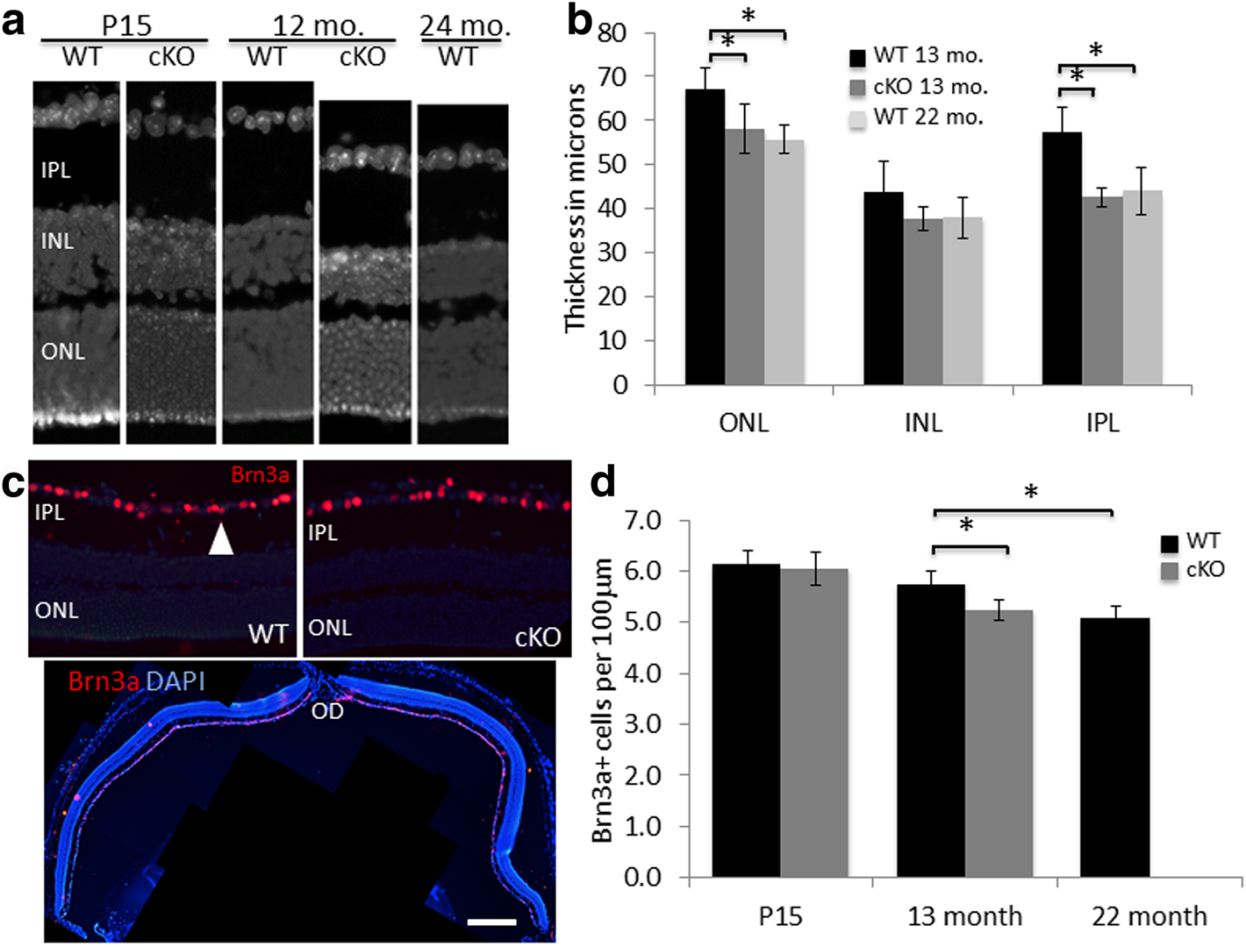
Retinal degeneration evident at 12–14 months of age in XBP1 fl/fl; Chx10-cre compared to wild type. **a** DAPI stained cryosections of XBP1 fl/fl (WT) and XBP1 fl/fl; Chx10-Cre (cKO) retina at P15 and 12 months, and a WT cryosection at 24 months, that closely represent the average retinal layers for each genotype and age. **b** Graph of measurements of retinal layers showing statistically significant differences in the outer nuclear layer (ONL, *p* < 0.02) and inner plexiform layer (IPL, *p* < 0.001) between WT (*n* = 7) and XBP1 cKO (*n* = 5) at 12–14 months (13 mo.). Graph also depicts statistically significant differences in the outer nuclear layer (ONL, *p* < 0.01) and inner plexiform layer (IPL, *p* < 0.01) between WT at 12–14 months and WT at 20–24 months (22 mo., *n* = 4). **c** Antibodies against Brn3a (red, arrowhead) label a majority of RGCs in WT (upper left) and XBP1 cKO (upper right) at 12–14 months. Sections are counterstained with DAPI (blue). Low magnification of a cryosection through the optic disk (OD, bottom). **d** Graphs show the number of Brn3a-positive cells within the ganglion cell layer at P15 (data from Fig. 1h), 13 months, and at 22 months. There is a statistically significant decrease (*p* < 0.01) in Brn3a-positive cells at age 13 months between WT (black, *n* = 6) and XBP1 cKO (grey, *n* = 5). In addition, there is a statistically significant decrease (*p* < 0.01) in Brn3a-positive cells between WT at age 13 months and at age 22 months (*n* = 4). INL, inner nuclear layer. Scale bar = 40 μm in A and 60 μm in C, upper, and 350 μm in C, lower

We sought to determine if the decline in ERG responses in XBP1 cKO between 8 months and 12 months of age was due to deterioration of the neural retina. As described above, XBP1 cKO retinas are indistinguishable from WT at P15 and functionally identical in the ERG through eight months of age. Using the same analyses on 12–14 month retinas as we used for P15 retinas, we find multiple significant differences manifest in XBP1 cKO retina compared to WT. Our measurements of retinal layers and specific cell type populations reveal significantly more deterioration in the XBP1 cKO retina than in age-matched WT retina after one year of age. Remarkably, XBP1 retinal structure at 12–14 months is essentially indistinguishable from WT retina at 22 months of age (Fig. 4).

By 12–14 months of age in XBP1 cKO retina we find that the ONL and the inner plexiform layer (IPL) are significantly thinner in central retina (see Methods) compared to WT at this age and closely matched to WT at age 22 months (Fig. 4a and b). The inner nuclear layer (INL) in XBP1 cKO retina is also reduced to a somewhat greater extent than WT, though not statistically significant. Although the ONL is thinner in XBP1 cKO retina than in WT at 12–14 months, we find no difference in the number of DAPI-labeled nuclei in the ONL between genotypes (WT: 8.8 +/− 0.1 cells and XBP1 cKO: 8.7 +/− 0.5 cells per 10 μm ONL column).

Counts of Brn3a-positive cells within the GCL reveal significantly fewer Brn3a-positive cells in the XBP1 cKO retina compared to age-matched 12–14 month old WT retinas (Fig. 4c and d). These data demonstrate a naturally occurring 6% decline in Brn3a-positive cells from P15 to 12–14 months of age in WT retina. This decline is more than doubled in XBP1 cKO retina, with 14% fewer Brn3a-positive cells in 12–14 month XBP1 cKO retina compared to P15 XBP1 cKO retina. Again, we find the 12–14 month old XBP1 cKO retina closely resembles WT retina at 22 months. The 12–14 month XBP1 cKO has significantly fewer Brn3a-positive cells than 12–14 month old WT to a nearly identical extent as the 22 month old WT retina (Fig. 4d). We find no obvious difference between 12 month XBP1 cKO and WT in the number of MG at this age as determined by antibodies against the MG marker, GS, nor do we find any significant difference in Pax6-positive cells (Additional file 1: Figure S1).

### Increase in ectopic bipolar cell dendrites and synapses in 12–14 month-old XBP1 cKO retina

**Fig. 5:**
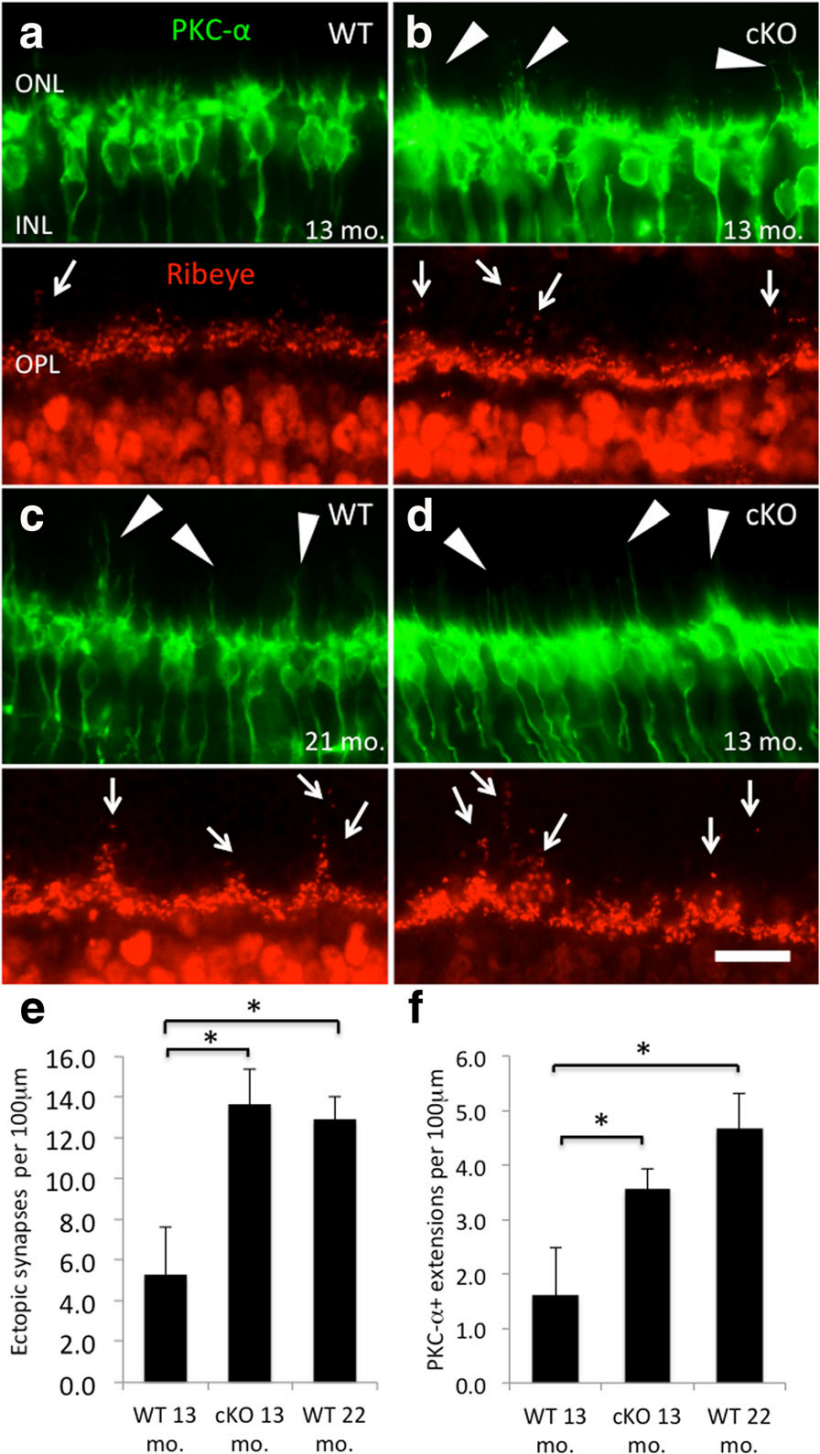
Bipolar cells in XBP1 fl/fl; Chx10-Cre retina extend dendrites into ONL and have more ectopic synapses than in age-matched WT. (**a** to **d**) Cryosections of retina labeled with antibodies against PKC-α (green), Ribeye (red) for XBP1 fl/fl (WT) at **a** 13 months and at **c** 21 months of age and XBP1 fl/fl; Chx10-cre positive (cKO; **b** and **d**) at 13 months of age. (**b** and **d**) XBP1 fl/fl; Chx10-Cre (cKO) at 13 months of age showing multiple extensions from bipolar cells into the outer nuclear layer (ONL; arrowheads) as well as the ectopic synapses outside the outer plexiform layer (OPL) in the ONL (arrows). **c** Similarly, WT retina at 21 months has multiple extensions from bipolar cells into ONL (arrowheads) and ectopic synapses in the ONL (arrows). **e** Graphs depict the number of ectopic synapses in the ONL. There are more than twice as many ectopic synapses in the XBP1 cKO (*n* = 4) than in WT (*n* = 5) at 13 months of age. Similarly, there are more than twice as many ectopic synapses in WT (*n* = 4) at age 22 months than at WT at age 13 months. **f** There are significantly more extensions from PKC-α positive bipolar cells into the ONL in XBP1 cKO compared to WT retina at 13 months. Similarly, there are significantly more extensions from PKC-α positive bipolar cells into the ONL in WT retina at 22 months of age compared to WT retina at 13 months of age. Scale bar = 20 μm. INL, inner nuclear layer. *, *p* < 0.05

Examination of bipolar cells and their synapses in the outer plexiform layer (OPL) reveals more retinal deterioration in the XBP1 cKO than in the WT retina (Fig. 5). We find a trend for a small reduction in the number of PKC-α positive rod bipolar cells in 12–14 month old XBP1 cKO retinas (11.5+/− 2 cells per 100 μm), compared to WT controls at 12–14 months (13.2+/− 2) and 22 month old WT (12.7+/− 0.6), though the result is not statistically significant. In contrast the morphology of bipolar cells and their synapses in XBP1 cKO retina differs significantly from WT at 12–14 months of age and closely resembles 22 month old WT retina. Antibodies against PKC-α, and Ribeye, a marker for ribbon synapses between photoreceptors and bipolar cells, reveals features of the 12–14 month old XBP1 cKO retina (Fig. 5b and d) that are more similar to very old (i.e. 24 months old) retina (Fig. 5c) than to age-matched WT retina (Fig. 5a). Specifically, the number of ectopic synapses (i.e., Ribeye-positive puncta within the ONL) between photoreceptors and bipolar cells in XBP1 cKO retina is more than twice that of age-matched WT retina, but closely matching 22 month WT retina (Fig. 5e). Furthermore, though cell number is not different, we find more than twice the number of PKC-α-positive bipolar cell extensions into the ONL in XBP1 cKO retina than in age-matched WT retina, which again resembles 22 month old retina (Fig. 5f).

### Activation of microglia corresponds to discontinuous synaptic lamina in the IPL of XBP1 cKO mice

**Fig. 6:**
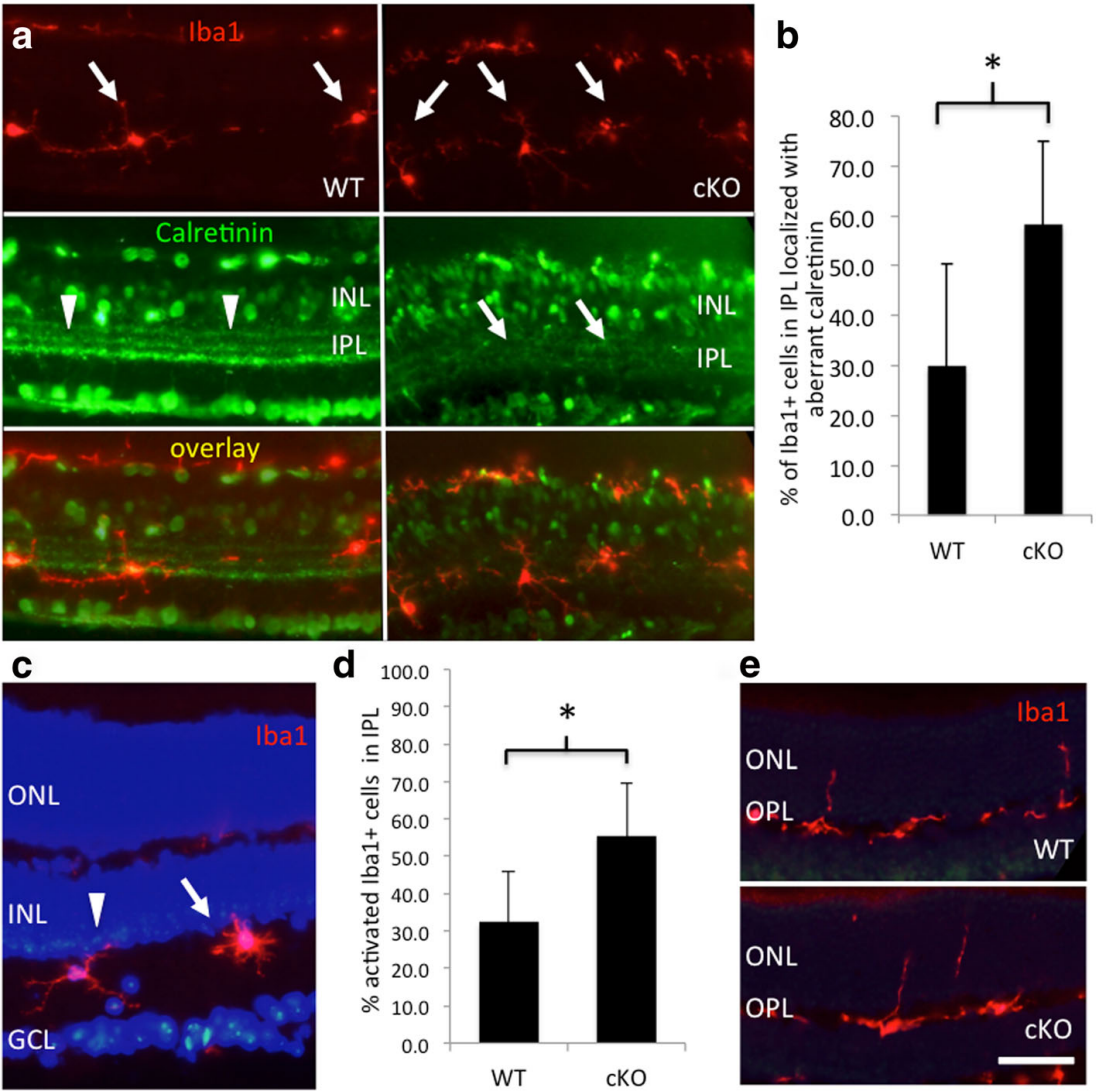
Iba1 positive cells in the inner plexiform layer correlate with calretinin discontinuities in the XBP1 fl/fl; Chx10-Cre retina. **a** Cryosections of 12–14 month retina labeled with the Iba1 antibody (red) and anti-calretinin (green). Iba1-positive cells within the inner plexiform layer (IPL) in WT and XBP1 fl/fl; Chx10-Cre (cKO) show several distinct features. Calretinin labeling within the IPL labels three defined strata (arrowheads). Areas of discontinuity (arrows) are evident. **b** Iba1-postive cells correlate with areas of calretinin discontinuity twice as frequently (*p* < 0.03) in XBP1 cKO (*n* = 5 mice, 66 Iba1+ cells) than in WT (*n* = 5 mice, 67 Iba1+ cells) retina. (**c** and **d**). Additionally, Iba1-positive cells are characterized as having either amoeboid morphology indicating activated microglia (arrow) or ramified morphology indicating resting microglia (arrowhead). There are nearly twice as many (*p* < 0.01) activated Iba1-positive cells in the IPL of XBP1 cKO retina (*n* = 7 mice, 103 cells) compared to WT (*n* = 7 mice, 98 cells). **e** In contrast, in the outer plexiform layer (OPL), we find no obvious differences in Iba1-positive cell number (WT and cKO, 2 Iba1+ cells per 100 μm) and extensions into outer nuclear layer (ONL; WT, 0.55 and cKO 0.49 extensions per cell) (*n* = 7 mice, 128 cells) and XBP1 cKO (*n* = 7 mice, 168 cells). GCL, ganglion cell layer. Scale bar = 25 μm in A, 50 μm in **c** and **e**

A detailed analysis of synaptic lamina in the IPL reveals that the three calretinin-positive synaptic laminae appear discontinuous and unorganized in many areas in 12–14 month XBP1 cKO retinas (Fig. 6). Further analyses demonstrate that Iba1-positive cells (microglia) within the IPL commonly are associated with unorganized areas of calretinin labeling, suggesting these areas contain deteriorated synapses (Fig. 6b). The association of Iba1-positive cells with unorganized calretinin labeling within the IPL is significantly greater in XBP1 cKO retina than in WT retina. In XBP1 cKO retina, more than 58% of Iba1-positive cells are localized to areas of unorganized calretinin labeling, nearly twice the percentage found in WT retina (30%; Fig. 6b). Additionally, in WT IPL nearly one-third (32%) of Iba1-positive cells have morphology indicative of activation (i.e. short, thick processes, and a rounded cell body), however, in the XBP1 cKO IPL over half (56%) of Iba1-positive cells have activated morphology (Fig. 6c and d). Thus, the synapses in the IPL, primarily synapses between bipolar cells and RGCs, appear unorganized more often in the XBP1 cKO than in WT and Iba1-positive cells in the IPL are about twice as likely to be associated with disorganized lamina and twice as likely to be in an activated state in 12–14 month old retinas. In contrast, most Iba1-positive cells in the IPL of WT mice have small cell bodies and elongated processes and are not associated with discontinuous calretinin-positive synaptic lamina.

In contrast to the IPL, the microglial population within the OPL of XBP1 cKO retina is not significantly different from that of WT controls. We find no difference in the prevalence of extensions into the ONL between WT and XBP1 cKO (Fig. 6e). Further, the average lengths of these extensions are not different (WT: 12.9 +/− 3.2 μm cKO:12.4 +/− 3.4 μm). Thus, the changes in microglial population and morphology appear to be limited to the IPL in XBP1 cKO retina.

### Defective glycolysis in the XBP1 cKO retina

**Fig. 7:**
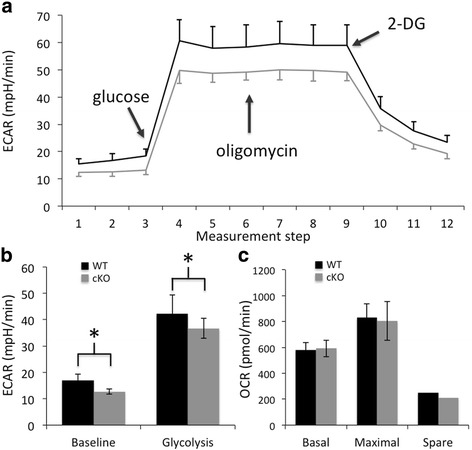
Glycolysis is less efficient in XBP1 fl/fl; Chx10-Cre retina than in age-matched wild type retina. **a** Graph depicting the normalized extracellular acidification rate (ECAR) at twelve measurement points for WT (black, *n* = 5 retinas, 18 explants) and XBP1 fl/fl; Chx10-Cre (cKO, grey, *n* = 5 retinas, 18 explants) retinal explants, aged 12–15 months throughout a glycolysis stress test in a Seahorse extracellular flux analyzer. Injections of glucose, oligomycin, and 2-D-glucose (2-DG) were made as indicated. **b** Baseline ECAR is significantly (*p* < 0.01) reduced in XBP1 cKO compared to WT, as is peak glycolysis (*p* < 0.04). A mitochondrial stress test reveals no differences in oxygen consumption rate (OCR) between retinal explants from 12 to 15 month old WT (*n* = 4 retinas, 18 explants) and XBP1 cKO (*n* = 4 retinas, 18 explants) mice

A hallmark of aging is deregulated nutrient sensing leading to metabolic alterations [16]. We used retinal explants from XBP1 cKO and WT mice aged 12–15 months in metabolic stress tests. We find that XBP1 cKO retinal explants have a baseline metabolism about 25% lower than WT explants, as measured by extracellular acidification rate (ECAR) in a glycolysis stress test using a Seahorse Extracellular Flux Analyzer (Fig. 7). Furthermore, the maximal glycolytic response in XBP1 cKO retinal explants is decreased 13% compared to WT, when measured from baseline (Fig. 7b). The addition of oligomycin to inhibit oxidative phosphorylation and push cells towards glycolysis has no measurable effect on ECAR in this assay, indicating that glycolysis is the primary metabolic mechanism in retina. Consistent with this, we find no difference in the oxygen consumption rate (OCR), a measure of mitochondrial respiration, between 12 and 15 month XBP1 cKO and WT explants in a mitochondrial stress test (Fig. 7c). Thus, the primary source of metabolism in retina, glycolysis, is decreased at a baseline level in XBP1 cKO retinal explants compared to WT and maximal glycolysis is also reduced in the XBP1 cKO compared to WT at 12–15 months of age.

## Discussion

In the present study, we for the first time investigate the long-term effects of XBP1 deficiency on retinal neuronal development and maintenance. We demonstrate that in aged mouse retinas, the ER stress response, as measured by the activation of XBP1, is less effective as in the younger ones. Furthermore, very old WT retinas (20–24 months) have a lower level of XBP1s than young adult (4 months) WT retina, but a higher level of CHOP. These data suggest an age-related deficit in the IRE1/XBP1 signaling pathway in the retina. Thus, we examined the consequences of a lack of XBP1 in retinal cells using a XBP1 cKO mouse line. We find that the retinas of XBP1 cKO mice at 12–14 months of age demonstrate structural degeneration that closely resembles WT mice aged 20 months or older, and display functional deficits that are not yet present in age-matched WT mice. Interestingly, no significant defects in retinal structure or function were identified in the cKO mice through the age of eight months, suggesting that the lack of XBP1 in a subset of retinal cells does not affect retinal development. However, the deficit in XBP1 may compromise the retinal responses to natural and chronic stresses over a long time period (i.e. 12 months), which may subtly but cumulatively affect cellular metabolism and synaptic transmission [5, 6, 16], consequently resulting in accelerated functional decline, metabolic deficits, and structural deterioration.

Our data indicate that the deletion of XBP1 in retinal progenitor cells, driven by Chx10 expression, does not affect retinal neuronal development. This finding does seem surprising, given that previous studies have reported an important role of XBP1 in brain-derived neurotrophic factor (BDNF)-mediated neurite outgrowth [17, 18] as well as in axon regeneration in *C. elegans* [19] and in Drosophila [20]. Yet currently there is no direct evidence to support that lack of XBP1 results in significant structural defects in the development of central nervous system. It is perhaps logical to speculate that in addition to XBP1, other UPR molecules, e.g. ATF6, that orchestrate the adaptive response to physiological stresses, may play an equally important, and compensatory, role in regulation of neuronal development and function. This is in part supported by several recent reports identifying mutations of human ATF6 that interrupt the proper activation of this UPR molecule during ER stress contributing to the development of achromatopsia, an autosomal recessive retinal disease characterized by cone dysfunction [21, 22]. Interestingly, we only observed a modest and insignificant increase in ATF6 level in the retina of XBP1 cKO mice. Whether this change, or the changes in other molecules, could exert a compensatory effect in regulation of the adaptive UPR during retinal development in the XBP1 cKO mice remains to be elucidated.

Despite the negative finding in retinal development, our data strongly suggest an essential and beneficial role of XBP1 in protecting retinal neurons against age-related degeneration. At 12–14 months of age, XBP1 cKO mice demonstrate retinal thinning, indicative of cell loss or shrinkage in the IPL and ONL, and ganglion cell loss in the GCL. At this point we cannot differentiate between an appropriate expansion of the IPL after P15, followed by an accelerated reduction in the XBP1 cKO, versus a simple lack of the IPL expansion observed in WT from P15 to 12–14 months. Importantly, we find that bipolar cells in the year-old XBP1 cKO have more than twice as many dendritic extensions into the ONL and more than twice as many ectopic synapses in the ONL, compared to age-matched WT. The increase in bipolar cell dendritic extensions into ONL is strikingly similar to what occurs in very old (i.e. 24 month) mice [2, 3]. A previous study [4] shows that bipolar extensions into ONL are following retracted photoreceptor axons. We speculate that in XBP1 cKO mice, bipolar cell dysfunction, as indicated by reduction in ERG b wave, could be the underlying mechanism by which bipolar-photoreceptor synapses break down, leading to axon retraction. Interestingly, we did not observe any significant difference in the number of ONL neurons nor in PKC-α labeled bipolar cells in the retina of XBP1 cKO mice. This is consistent with an earlier report that aged mice do not have significantly fewer bipolar cells or rods than young mice [2]. Thus, chronic dysfunction of bipolar cells may result in aberrant bipolar extension and ectopic synapsis in aged XBP1 cKO mice.

Another interesting finding from our study supports a role of microglial activation in age-related neuronal degeneration. We demonstrate a significant increase in disrupted calretinin-positive synaptic lamina colocalized with Iba1-positive microglia in the IPL of XBP1 cKO retina. Furthermore, the total number of activated Iba1-positive microglia in the IPL was also increased in the cKO mice. One important function of microglia is to monitor and eliminate unhealthy synapses [23]. We propose that synaptic dysfunction in the XBP1 cKO retina may trigger microglial activation to eliminate damaged synapsis resulting in discontinuities in the trilaminar calretinin labeling, which was observed only in mice at about 1 year of age or older. Another possibility is that changes in Müller cells, which can be potentially affected by Chx10-driven XBP1 deletion, may influence microglia activation. A close interaction between microglial activation and Müller cell abnormality has been demonstrated previously [24]. Although our preliminary study shows no significant change in the morphology of Müller cells, whether changes in the metabolism and function of these cells contribute to microglial activation in the cKO retina will be pursued in future studies. Nevertheless, the potential influence of XBP1 in retinal cells on microglial activation is of great interest, as the increased number of activated microglia has also been observed in aged human retinas [25]. Uncontrolled microglial activation, through producing pro-inflammatory factors and interacting with Müller glia and retinal neurons, may exacerbate neuronal injury eventually resulting in degeneration of the retina with aging.

Finally, our study identifies a potential role of XBP1 in regulation of retinal metabolism, which could in part contribute to the premature decline in retinal function and structural deterioration in XBP1 cKO mice. Specifically, our data imply a more prominent role for XBP1 in the regulation and process of glycolysis. This finding is in line with a recent report demonstrating that silencing XBP1 in glioma cells inhibits glycolysis resulting in reduced ATP production and decreased cancer cell survival [26]. Long-term activation of the IRE1 signaling has also been shown to reduce glucose metabolism and mitochondrial function; however, it is unclear whether this effect is mediated by XBP1 [27]. Intriguingly, deficiency of XBP1 has been shown to induce an overactivation of IRE1, contributing to enhanced inflammation and apoptosis [28]. Whether XBP1 deficiency influences IRE1 activation, which in turn reduces glycolysis in the cKO retina, is yet to be determined. In addition, although we observe no significant difference in mitochondrial function between the XBP1 cKO retina and the WT, a more detailed analysis of mitochondrial function in non-photoreceptor cells is warranted. In line with our findings, a study reports that mice carrying mutations in AMPK or LKB1, an upstream activator of AMPK, have a similar bipolar cell phenotype, as observed in our XBP1 cKO mice, attributed to retraction of rod axons [4]. Furthermore, Lkb1 fl/fl; Chx10 Cre mice also demonstrate a large decline in the ERG response at 8–9 months of age [4]. These observations collectively suggest a critical role of metabolic disturbance in the development of age-related retinal neurodegeneration. In addition, our data show that aged WT retina has an increased expression of CHOP, further supporting a potential role of long-term chronic ER stress in retinal aging. Whether increased CHOP expression contributes to age-related retinal structural and functional deterioration should also be evaluated in the future.

## Conclusions

Aged retina demonstrates reduced basal level and defective activation of XBP1 in response to ER stress. Deletion of XBP1 in a subset of retinal cells accelerates the age-related decline in retinal function and neurodegeneration. These effects are likely, at least in part, mediated by disturbed glycolysis and increased microglial activation. Given the difficulty in nervous system repair we suggest preservative intervention in late adulthood is the best path to long-term functional retention at advanced ages. Understanding the molecular basis of XBP1 and the UPR in regulation of age-related stresses in retinal neurons would facilitate the development of new approaches to promote the preservation of visual function.

## Additional files

Additional file 1: **Figure S1.** Multiple retinal markers reveal no differences between WT and XBP1 cKO at P15 or 12 months of age. Cryosections of retina from P15 or 12 month old wild type (WT) and XBP1 fl/fl; Chx10-cre (cKO) mice immunolabeled with antibodies against the listed proteins. (A) At P15 there is no difference in the appearance of labeling for calbindin, a horizontal cell marker, between XBP1 cKO and WT. (B and C) There are no differences in labeling for Pax6 at (B) P15 or (C) 12 months between XBP1 cKO and WT retinas. (D) Double labeling for the bipolar cell marker, PKC-α, and the Ribeye antibody appears identical between XBP1 cKO and WT at P15. (E and F) We see no differences between XBP1 cKO and WT in staining for the MG marker, glutamine synthetase (GS) at P15 or 12 months of age. GCL, ganglion cell layer; INL, inner nuclear layer; ONL, outer nuclear layer. Scale bar = 40 μm. (TIFF 1571 kb)

Additional file 2: **Figure S2.** No differences in ERG responses between WT and XBP1 cKO mice at 10 weeks of age or in implicit times at any age. (A) Graph of the a-wave and b-wave responses for a transient, light-adapted ERG for XBP1 fl/fl (WT, black, *n* = 5) and XBP1 fl/fl; Chx10-Cre (cKO, grey, *n* = 5) at 10 weeks of age shows no differences in the amplitude of the a-wave or b-wave. (B) In addition, the implicit times for b-wave onset are not different for WT and XBP1 cKO in transient, light adapted ERG responses at any age measured. (C and D) Similarly, implicit times for the a-wave and b-wave are not different for the 10-step dark adapted ERG responses between (C) 6–8 month old WT and XBP1 cKO mice nor between (D) 12–14 month old WT and XBP1 cKO mice. (TIFF 449 kb)

## Abbreviations

cKO: conditional knock-out
ECAR: extracellular acidification rate
ER: endoplasmic reticulum
ERG: electroretinogram
GCL: ganglion cell layer
INL: inner nuclear layer
IPL: inner plexiform layer
OCR: oxygen consumption rate
ONL: outer nuclear layer
OPL: outer plexiform layer
P: postnatal day
RGC: retinal ganglion cell
UPR: Unfolded Protein Response
WT: wild type
