# Supplementary figures and images for: Loss of XBP1 accelerates age-related decline in retinal function and neurodegeneration
Source: Mol Neurodegener. 2018 Apr 4;13:16. doi: 10.1186/s13024-018-0250-z (PMC5883257; doi:10.1186/s13024-018-0250-z)

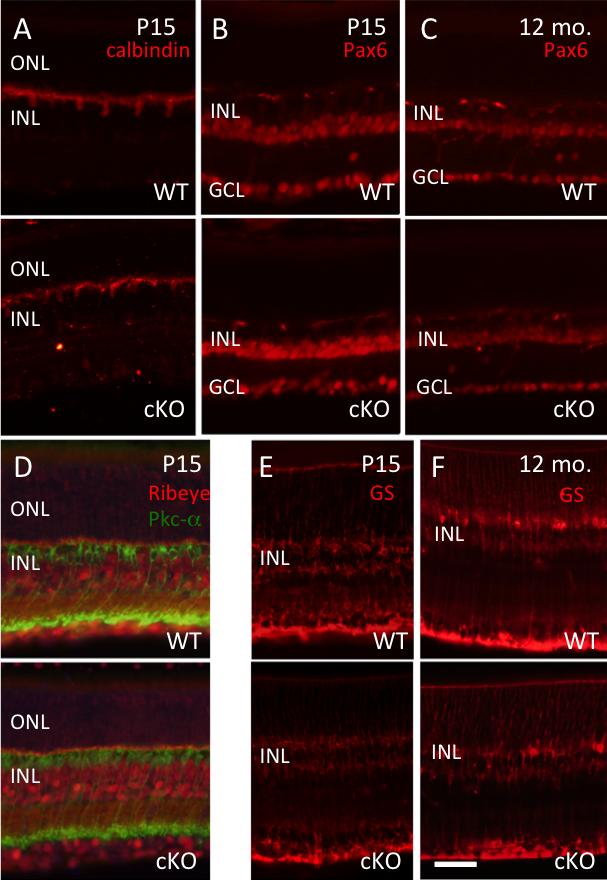

Supplement: Supplementary file 1 — Figure S1. Multiple retinal markers reveal no differences between WT and XBP1 cKO at P15 or 12 months of age. Cryosections of retina from P15 or 12 month old wild type (WT) and XBP1 fl/fl; Chx10-cre (cKO) mice immunolabeled with antibodies against the listed proteins. (A) At P15 there is no difference in the appearance of labeling for calbindin, a horizontal cell marker, between XBP1 cKO and WT. (B and C) There are no differences in labeling for Pax6 at (B) P15 or (C) 12 months between XBP1 cKO and WT retinas. (D) Double labeling for the bipolar cell marker, PKC-α, and the Ribeye antibody appears identical between XBP1 cKO and WT at P15. (E and F) We see no differences between XBP1 cKO and WT in staining for the MG marker, glutamine synthetase (GS) at P15 or 12 months of age. GCL, ganglion cell layer; INL, inner nuclear layer; ONL, outer nuclear layer. Scale bar = 40 μm. (TIFF 1571 kb) [file 13024_2018_250_MOESM1_ESM.tif]

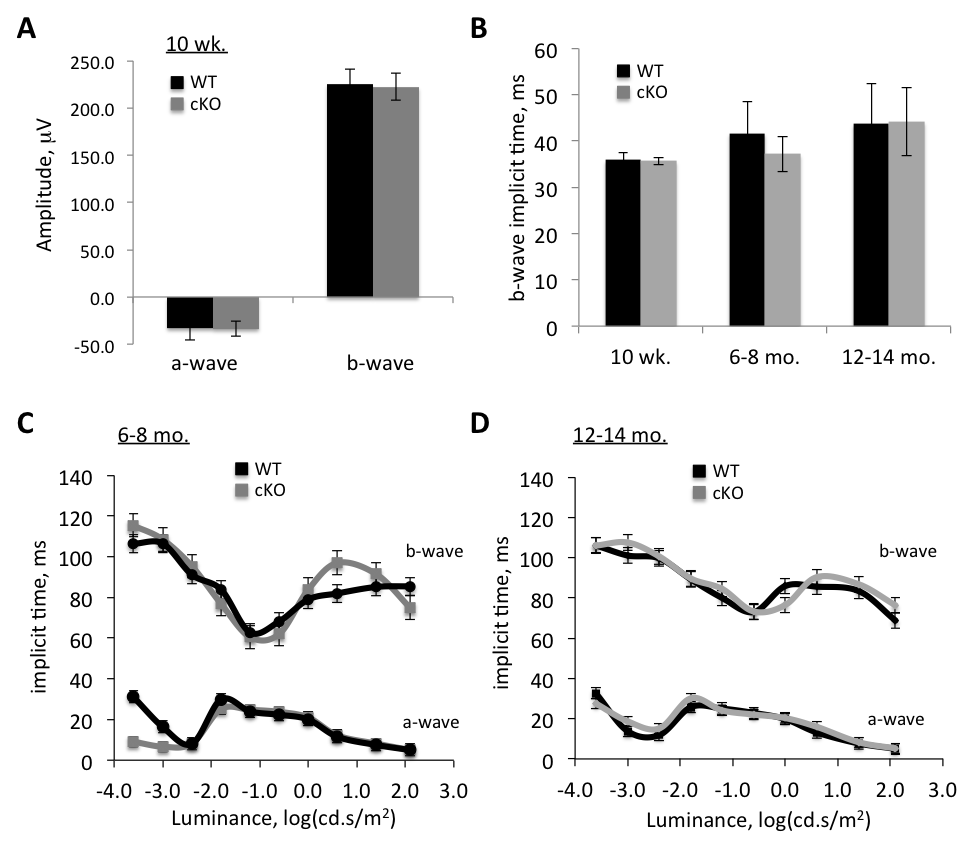

Supplement: Supplementary file 2 — Figure S2. No differences in ERG responses between WT and XBP1 cKO mice at 10 weeks of age or in implicit times at any age. (A) Graph of the a-wave and b-wave responses for a transient, light-adapted ERG for XBP1 fl/fl (WT, black, n = 5) and XBP1 fl/fl; Chx10-Cre (cKO, grey, n = 5) at 10 weeks of age shows no differences in the amplitude of the a-wave or b-wave. (B) In addition, the implicit times for b-wave onset are not different for WT and XBP1 cKO in transient, light adapted ERG responses at any age measured. (C and D) Similarly, implicit times for the a-wave and b-wave are not different for the 10-step dark adapted ERG responses between (C) 6–8 month old WT and XBP1 cKO mice nor between (D) 12–14 month old WT and XBP1 cKO mice. (TIFF 449 kb) [file 13024_2018_250_MOESM2_ESM.tif]
